# Study on Bearing Strength and Failure Modes of Single Bolted Joint Carbon/Epoxy Composite Materials

**DOI:** 10.3390/polym16060847

**Published:** 2024-03-19

**Authors:** Sang Min Park, Jin Hwan Jeon, Won Jong Choi

**Affiliations:** 1 Department of Smart Air Mobility, Korea Aerospace University, Goyang 10540, Republic of Korea; 2 Department of Materials Science & Engineering, Korea Aerospace University, Goyang 10540, Republic of Korea

**Keywords:** CFRP, single bolted joint, single-hole laminates, bearing strength, w/D ratio, e/D ratio

## Abstract

The growth of the Urban Air Mobility (UAM) industry emphasizes the need for considerable study into assembly procedures and dependability to guarantee its effective integration into air transport networks. In this context, this study seeks to evaluate the mechanical characteristics of bolted joint Carbon Fiber Reinforced Plastic (CFRP), with a particular emphasis on bearing strength. By altering the w/D (specimen width to hole diameter) and e/D (distance between hole center and specimen end to hole diameter) ratios, the study investigates how edge and end distances affect material performance. The study discovered a shift from tension to bearing failure at w/D ratios of 4.0, with maximum bearing strength decreases of 90.50% and 69.96% compared to full bearing failure. Similarly, for e/D ratios of 1.5, 2.0, and 3.0, transitioning from shear to bearing failure at 2.0 resulted in maximum bearing strength losses of 94.90% and 75.96%, respectively. Maintaining a w/D ratio of at least 6.0 and an e/D ratio of at least 3.0 is critical for maintaining maximum performance and stability in CFRP structure design.

## 1. Introduction

Composite materials are widely sought after in a variety of industries due to their superior mechanical qualities, which include a high strength-to-weight ratio and a low stiffness-to-weight ratio, allowing for the creation of lightweight solutions [1,2,3,4,5,6,7]. They have several uses in transportation, including the aerospace, automotive, and marine sectors, where they make up more than half of all aircraft structural components [8,9,10,11,12,13,14]. Furthermore, they are used in the automobile and sports industries [15,16,17,18,19,20]. As the UAM industry continues to grow, there is a rising need to understand the bearing strength of CFRP bolted joints.

Understanding the bearing strength of bolted joints is critical for closing the gap between the intrinsic qualities of composite materials and their practical applications. This promotes smoother integration across sectors by allowing for a more exact prediction of bearing strength under specific situations. Furthermore, the retrieval and assessment of such data are likely to have a substantial influence on the creation of new composite materials and the improvement of existing material performance. This will enable researchers to investigate the properties of innovative materials and develop products that surpass existing ones. The collection and analysis of data on the bearing strength of CFRP bolted joints is driving the development of novel composite materials and technologies.

Recent research has made important advances in our knowledge of composite material behavior and the significance of bolted joints, including studies on bearing strength using finite element analysis and using rivets and countersunk bolts in joints. Studies on structural parameters, such as the w/D and e/D ratios, have also provided important insights into designing new goods and technology [21]. 

For example, Guo qiang Gao et al. used progressive damage modeling and simulations to forecast the strength of aircraft bolted joints in composite laminates, which improved design, analysis, and certification efficiency [22]. Long Yan et al. investigated the influence of interference fit sizes on damage in CFRP hybrid bonded–bolted joints and discovered matrix compression failure as the most common failure mechanism [23]. Gang Liu et al. created an asymptotic damage model to predict failure behavior in complicated composite laminated bolted joints and verified it with experimental results [24]. Jeong Sik Kwon et al. proposed a complete strategy for mechanically fastened composite laminate joints, which included progressive failure analysis to predict test loads and displacement [25]. Xin Li et al. studied CF/BMI composites in single-lap, single-bolt joints and discovered delamination as the predominant non-catastrophic failure mechanism in bearing failure [26]. 

Rivet joint research is ongoing. Zhenchao Qi et al. are enhancing ductility in aircraft CFRP structures using a current-assisted method [27]. Mauricio Torres-Arellano et al. are studying the mechanical performance in resin transfer molded joints [28]. Natascha Z. Borba et al. are investigating thermo-mechanical changes and their correlation with mechanical properties in friction-riveted joints [29]. 

Guido Di Bella et al. investigated orbital riveting as an effective connecting method. At the same time, Lu-ana Pereira Dalla Mariga et al. investigated the impact of geometrical tolerance on single-lap composite joints riveted with two fasteners [30,31]. Countersunk bolts have been investigated for the correction of drilling-induced faults, as well as the influence of stacking sequences on the bearing strength of composite bolted joints [32,33,34]. Manasi Palwankar et al. demonstrated a revised fixture for bolted composite joint testing that can support countersunk fasteners [35]. Studies have also looked at the environmental impact of bolted joints, corrosion-resistant alternatives, bearing performance in harsh conditions, and the effects of thermal exposure and seawater aging on bearing strength [36,37,38,39,40,41,42]. 

Dong-Uk Kim et al. and D. Sivakumar et al. examined the mechanical bearing strength and failure mechanisms of composite-to-metal joining structures connected by mechanically secured joints, demonstrating composites’ potential as a replacement for steel in ships and offshore constructions [43,44]. Furthermore, Hassan Al-shahrani et al., Z. Sajid et al., Anyang Wang et al., Jing-chao Wei et al., Junshan Hu et al., Andrew N. Dickson et al., Isha Paliwal et al., and Liyuan Qu et al. have made significant contributions to our understanding of bolted joint behavior in composite materials, providing valuable insights for the development of innovative products and technologies [45,46,47,48,49,50,51].

As a result, this study is significant because it improves our understanding of the structural behavior of bolted joints and helps us develop optimal designs and manufacturing procedures while considering variations in bearing strength caused by the w/D and e/D ratios. A detailed evaluation of bearing strength at different w/D and e/D ratios will propel improvements in aerospace and other industries that use composite materials, resulting in safer and more efficient products and technologies. Furthermore, this study has the potential to fill knowledge gaps and provide new insights, benefiting both academia and business. Finally, the study’s findings might have a substantial impact on technological advances, resulting in improved structural designs and material developments. Thus, research into w/D and e/D ratios has practical applications and significant implications.

## 2. Test Materials and Methods

### 2.1. Test Materials 

The specimens in this investigation were made from CYCOM^®^ 5320-1 T650-35 6K (Solvay, Alpharetta, GA, USA) prepreg, a unidirectional weave fabric impregnated with a 350 °F (177 °C) cure epoxy resin system. The manufacturer’s technical data sheet (TDS) specifies that this prepreg is acceptable for use in the vacuum bag only (VBO), autoclave, and prepreg compression molding (PCM) processes. The specimens for this investigation were created utilizing the VBO technique [52]. 

### 2.2. Specimen Fabrication

**Figure 1:**
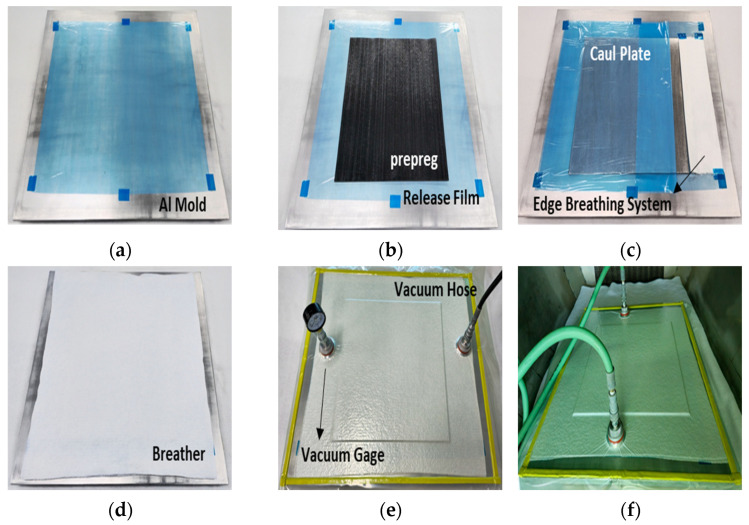
The manufacturing process of the bolted joint specimen through the VBO process. (**a**) Attachment of release film after mold cleaning, (**b**) stacking of prepreg ([+45/0/-45/90]_2S_) on the release film, (**c**) fixing of the edge breathing system made of glass fabric on the side, (**d**) stacking of the caul plate, release film, and breather on the prepreg, (**e**) conducting a leak test after vacuum bagging, and (**f**) placing the specimen in an oven and curing.

Figure 1 illustrates the panel construction procedure for specimen manufacturing. It involved the following steps: (1) An Al Mold (0.250”, Al 60xx) was made, sanded to 2000 grit or higher, and cleaned with Methyl Ethyl Ketone. A release film was applied to the Al mold (Figure 1a). (2) The prepreg was set out on the Al mold using the laboratory conditions recommended in ASTM D5229 [53]. The layup pattern was tuned to quasi-isotropic ([+45/0/-45/90]_2S_) for comparison, with the mechanical properties obtained by NCAMP (National Center for Advanced Materials Performance) shown in Figure 1b [54]. (3) After completing the prepreg stack, a caul plate (0.075” Al 60xx) and another release film were put on top. The edge breathing system was applied to the side of the prepreg stack using glass fabric (Style 1542) with a width of 3 inches (Figure 1c). (4) Vacuum pressure was applied using a breather and a vacuum bag with sealing tape. After disconnecting the hose following vacuum application, a leak test of 1 inHg or greater was performed for 5 min. A 30-min leak test was performed in this investigation, and no leaks were found (Figure 1d,e). (5) After the leak test, the mold was placed in an air circulation oven to cure. Airtech International Inc. provided all the process materials utilized in this study, including the vacuum bag, breather, release film, sealant tape, tape, and squeezer. The materials were carefully selected to enable effective vacuum bagging and sealing and to produce the breather system required for the VBO process.

**Figure 2:**
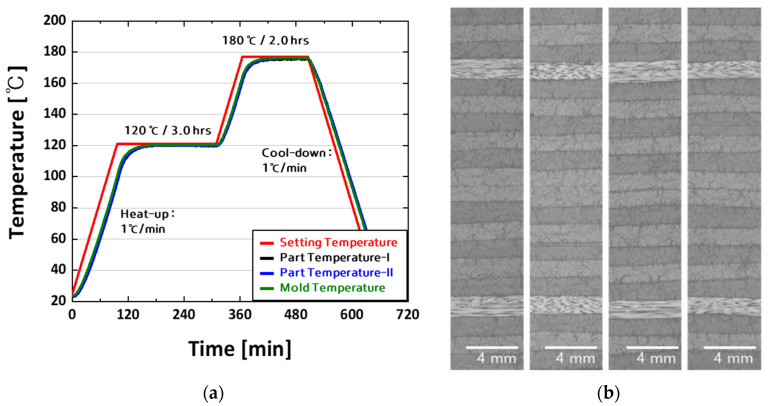
Cure cycle and internal optical microscopy (OM) images of the flat panel: (**a**) cure cycle “C” used for panel fabrication (as described in NCAMP). (**b**) OM image of the panel fabricated using the prescribed cure cycle.

Figure 2a shows the NCAMP cure cycle “C”, previously discussed in this work, and the actual temperature recorded within the panel using thermocouples. The temperature profiling results obtained via thermocouples indicate that the temperature deviation across the entire panel is within 1.0 °C. This means there was no substantial temperature variation inside the panel during the experiment. The temperature data for all panels used in the test were consistently within acceptable levels, and any panel that did not match the stipulated criteria was removed from the production process.

Figure 2b shows the inner side of the panel, where the manufacturing procedures mentioned in Figure 1 and Figure 2a were used, as seen via an optical microscope (OM). The specimen’s whole thickness direction was evaluated by OM, which demonstrated a perfect state, notably the lack of flaws during the curing cycle. These findings highlight the need for rigorous quality control processes and the production of high-quality specimens. The specimen’s defect-free character makes it ideal for bearing strength studies, increasing the reliability and validity of the experimental results. 

The curing cycle “C,” given by NCAMP, was used in this investigation to assess and compare the material compatibility for the bearing strength of the bolted joint CFRP specimen. This cure cycle consisted of six steps, including pre-cure vacuum hold, regulated heating, and precise holding periods, which helped achieve the composite material’s necessary mechanical characteristics. Thermocouples were strategically placed inside the composite panel and the Al mold to maintain temperature consistency during the cure cycle, with temperature data recorded at regular intervals. The results demonstrated temperature uniformity, ensuring the composite specimens were cured optimally and consistently.

### 2.3. Experimental Set-Up

**Figure 3:**
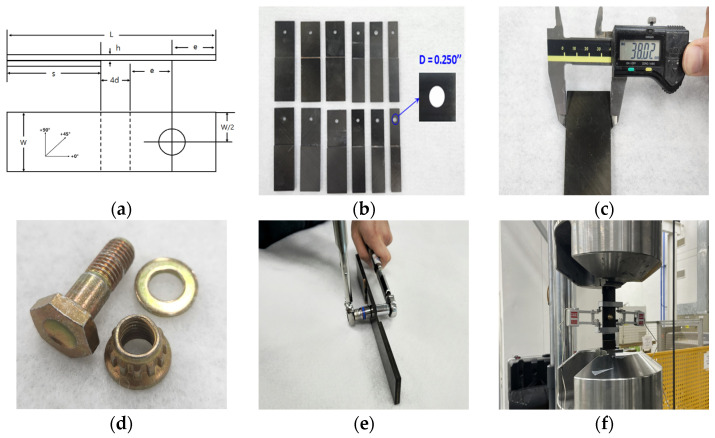
Bolted joint assembly and loading process of the specimen: (**a**) diagram of the single-shear, two-piece single-fastener test specimen; (**b**) specimen before bolted joint assembly; (**c**) precise dimension measurement of the specimen using vernier calipers and micrometers; (**d**) the fastener components (bolt, nut, washer); (**e**) fastening the two pieces of a specimen with a torque wrench ((3.4 ± 0.6) N-m); (**f**) installation of the extensometer and loading the specimen into the MTS 322.

The experiments in this study followed the ASTM D5961-17 standard test method. This method pertains to the bearing response of polymer matrix composite laminates. The single shear test without a support fixture, as outlined in procedure B of the standard, was conducted [55]. The test data obtained corresponded to the last version available at that time. The panels were fabricated utilizing precise mechanical machining using a low-vibration diamond cutter, resulting in the exact form represented in Figure 3a. The size of the specimens precisely adhered to the parameters provided in ASTM D5961, which were adjusted to the unique needs of this study [56]. Furthermore, hole machining was conducted while keeping the w/D and e/D ratios in mind to meet the study objectives (see Figure 3b). The specimens’ measurements, including width, length, thickness, and hole diameter, were measured five times with vernier calipers and micrometers (Figure 3c). After carefully analyzing the machining conditions and dimensions data, seven final test specimens were selected. The specific measurements of the chosen specimens are listed in Table 1.

Figure 3d shows real photos of the fasteners (bolt, nut, and washer) used in this test. Each fastener has a unique product name: bolt NAS6604-5, nut MS21084-04, and washer MS21206-4. To ensure accurate fastening, the fasteners were tightened using two torque wrenches to a torque of (3.4 ± 0.6) N-m, which is equivalent to 35% of the full clamp load (85 ± 5) specified in the NCAMP qualification material property data report (cam-rp-2013-002 rev a) shown in Figure 3e [55]. Following the attaching procedure, the specimens were placed on an MTS 322 (MTS Systems, Eden Prairie, MI, USA) testing equipment outfitted with extensometers. The tests were conducted at a crosshead speed of 2 mm/min [56].

### 2.4. Calculation of Bearing Strength

The applicable standard expressly specifies the calculating procedure for bearing strength, as demonstrated in this study. Equation (1) is used to determine the ultimate bearing strength ($F^{bru}$):

(1) $$F^{bru}=P^{max}/(k\times D\times h)$$

where $F^{bru}$ denotes the ultimate bearing strength (MPa). $P^{max}$ represents the maximum force applied to the specimen before failure (N). The value of k is the force per hole factor, which equals 1.0 for a single fastener. D represents the hole diameter (mm) and h is the specimen thickness (mm).

## 3. Test Results

### 3.1. Bearing Strength

**Figure 4:**
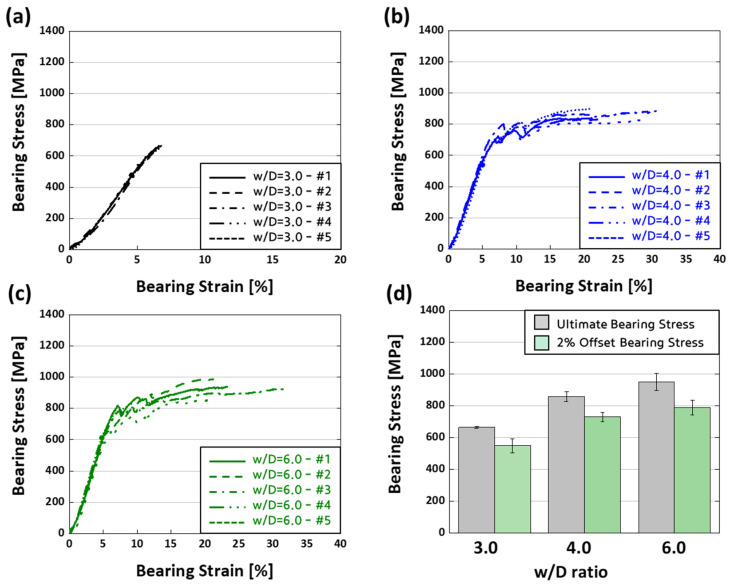
Comparative analysis of bearing strength for various w/D ratios: (**a**) w/D = 3.0; (**b**) w/D = 4.0; (**c**) w/D = 6.0; (**d**) scatter plot illustrating w/D ratios comparison.

**Figure 5:**
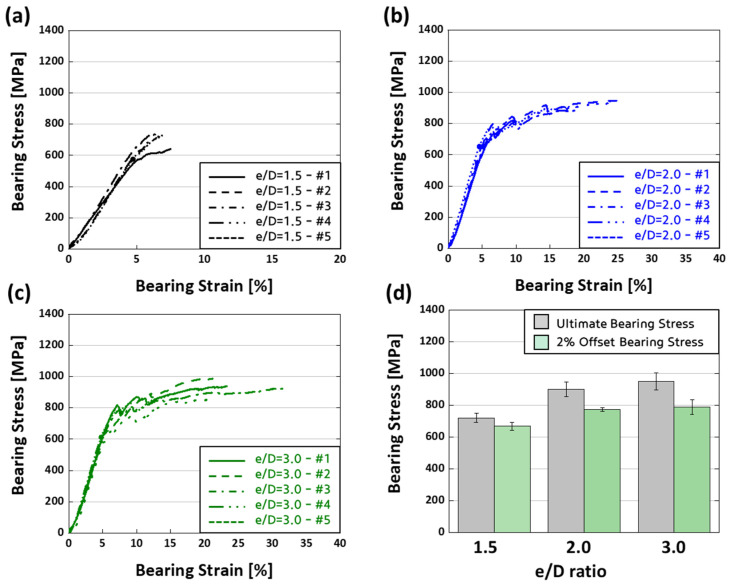
Comparative analysis of bearing strength for various e/D ratios: (**a**) e/D = 1.5; (**b**) e/D = 2.0; (**c**) e/D = 3.0; (**d**) scatter plot illustrating e/D ratios comparison.

Figure 4 and Figure 5 show the test findings from this investigation, which were conducted by ASTM D5961 procedure B [56]. Initially, seven specimens were assessed for each testing condition. However, following a thorough statistical analysis to eliminate outliers, only five specimens are shown in the figures. 

Figure 4 shows the bearing stress-strain curves organized by the w/D (width-to-diameter) ratio. Figure 4a,b illustrate the results for specimens with a w/D ratio of 3.0 and 4.0, respectively. Furthermore, Figure 4c gives further insights into material behavior by depicting a graph based on the findings of specimens with a w/D ratio of 3.0. Furthermore, Figure 4d shows a scatter plot that compares the ultimate bearing stress and the bearing stress computed using a 2% offset approach. 

The data from Figure 4 has been collated and presented numerically in Table 2, displaying the 2% offset bearing stress and ultimate bearing stress at various w/D ratios. The mean ± standard deviation of the data was reported to ensure accuracy and dependability, reflecting the observed variability in the experimental results.

Figure 5 shows the bearing stress–strain curves classified according to the edge-to-diameter (e/D) ratio. Figure 5a displays comprehensive results for specimens with an e/D ratio of 1.5, whereas Figure 5b shows findings for specimens with an e/D ratio of 2.0. Figure 5c also gives valuable insights into the material’s mechanical performance under various edge-to-diameter ratios by using a graph to depict the preliminary results of specimens with an e/D ratio of 3.0. In Figure 5d, a scatter plot allows for a complete comparison of the ultimate bearing stress and the bearing stress obtained using a 2% offset approach, providing a detailed investigation of the material’s performance under various loading situations. Table 3 was created to provide a thorough and exact representation of the data, including measured 2% offset bearing stress and ultimate bearing stress for various width-to-diameter (w/D) ratios. The data are provided in the form of a mean ± standard deviation to help ensure the findings are accurate and dependable.

### 3.2. Failure Mode

**Figure 6:**
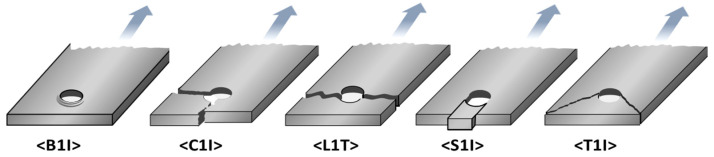
Bearing test failure codes with illustrations of common modes by ASTM D5961 standard.

According to the ASTM D5961 standard, failure modes are classified and visually represented as described in Figure 6. The classification system consists of three characteristics, each indicating a factor of the failure [56]. The first part corresponds to the failure type, with the following classifications: B for laminate bearing, C for laminate cleavage, L for laminate net tension, S for laminate shear-out, and T for laminate tear-out. The second denotes the failure area, with classifications including 1 for the 1st hole, 2 for the 2nd hole, B for both holes, 3 for the 1st fastener, 4 for the 2nd fastener, and F indicating both fasteners. Lastly, the third represents the failure location, with classifications such as B for laminate head side, N for laminated nut side, H for fastener head, C for fastener nut/collar, S for fastener shank, T for fastener thread, I for inapplicable, and U indicating unknown. For example, the code “B1I” in Figure 6 suggests that the failure type is a laminate bearing, the failure area is the 1st hole, and the failure location is inapplicable.

The tests based on the w/D ratio (w/D = 3.0, 4.0, 6.0) and the e/D ratio (e/D = 1.5, 2.0, 3.0) resulted in failure situations, which are described below: “L1I” Failure Mode: The failure type is laminate (lateral) net tension, the failure area is the first hole, and the failure location is inapplicable. “S1I” Failure Mode: The failure type is laminate shear-out, the failure area is the first hole, and the failure location is inapplicable. “B1I” Failure Mode: The failure type is a laminate bearing, the failure area is the first hole, and the failure location is inapplicable. “S3T” Failure Mode: The failure type is a laminate shear-out, the specific failure location is the 1st fastener, and the failure location is the fastener thread.

## 4. Discussion

### 4.1. Bearing Strength

Based on the experimental data reported in Section 3.1, a comparison of bearing strength yields interesting results. First, when the w/D ratio drops, bearing stress reduces significantly, which may be attributable to differences in load distribution and stress concentration around the hole. The load is evenly distributed in specimens with larger w/D ratios, resulting in bearing failure. However, when w/D ratios decline, stress concentration around the hole rises, increasing the likelihood of tensile failure, culminating in specimen weakening and failure [57]. Likewise, a drop in bearing stress is noticed when the e/D ratio decreases, owing to the shorter distance between the hole and the end of the specimen. As the e/D ratio drops, shear pressures inside the specimen become more noticeable, leading to shear failure [58]. 

**Figure 7:**
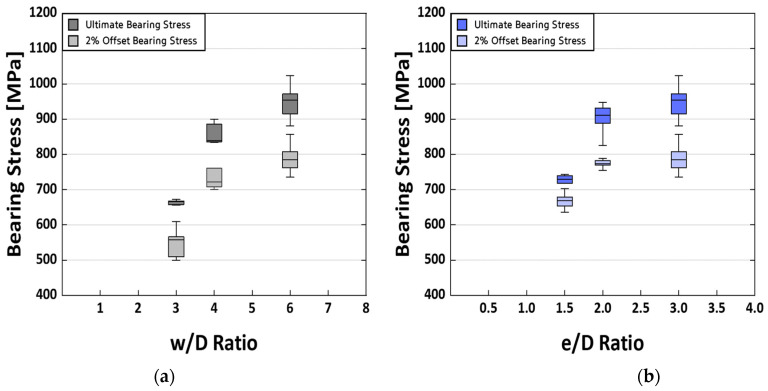
Boxplot comparisons of test results based on (**a**) w/D ratios and (**b**) e/D ratios.

Figure 7a shows how variations in the w/D ratio affect bearing stress behavior in specimens, with lower ratios resulting in a shift from bearing to tensile failure. The significant drop in bearing stress with decreasing w/D ratio is attributed to changes in load distribution and stress concentration around the hole. Higher w/D ratios cause even load distribution and attention on the bearing surface, which leads to bearing failure. As the w/D ratio decreases, the load distribution alters, increasing the likelihood of tensile failure owing to stress concentration around the hole [57,58,59].

Similarly, Figure 7b shows a reduction in bearing stress in relation to the e/D ratio. The typical e/D ratio of 3.0 is reduced to 2.0 or 1.5, resulting in a substantial decrease in bearing stress. Bearing failure occurs at e/D ratios greater than 2.5, whereas smaller ratios result in shear failure. As the e/D ratio falls, the holes move closer to the end of the specimen, lowering the distance from 0.750 inches to 0.375 inches. This shift causes shear stresses during testing, which then leads to fracture. As the e/D ratio drops, shear becomes the primary type of failure at the fracture site [24,60]. These results are also reflected in the final bearing strength.

When specimens with different w/D ratios are compared to the reference specimen with a w/D ratio of 6.0, the ultimate bearing strengths decline to 90.50% for w/D ratios of 4.0 and 69.96% for w/D ratios of 3.0. Similarly, when specimens with different e/D ratios are compared to the reference specimen with an e/D ratio of 3.0, the ultimate bearing strengths decline to 94.90% for an e/D ratio of 2.0 and 75.96% for an e/D ratio of 1.5. The observed reduction in bearing strength with changing w/D and e/D ratios is consistent with patterns described in earlier investigations.

In summary, as the w/D and e/D ratios decrease, the bearing strength decreases nonlinearly, consistent with the fracture mechanisms mentioned in Section 4.2. While the bearing strength is more sensitive to changes in the w/D ratio, the limited number of investigated w/D ratios prevents accurate representation using specific regression equations. The significant decrease in bearing stress with decreasing e/D ratios is attributed to the transition from bearing to shear failure, emphasizing the importance of optimizing geometric characteristics for individual applications while considering appropriate load conditions and failure modes [24].

### 4.2. Failure Mode

**Figure 8:**
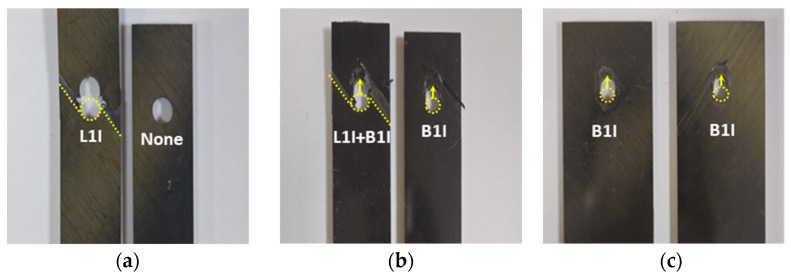

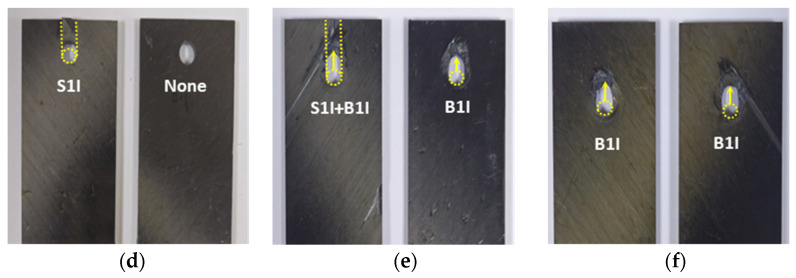
Failure modes of specimen holes captured using a microscope based on w/D and e/D ratios after testing: (**a**) w/D = 3.0; (**b**) w/D = 4.0; (**c**) w/D = 6.0; (**d**) e/D = 1.5; (**e**) e/D = 2.0; (**f**) e/D = 3.0.

As mentioned in Section 3.2, various failure mechanisms were identified in the test specimens based on the w/D and e/D ratios. Figure 8 illustrates the failure modes associated with each condition in this study, classified according to the ASTM D5961 standard [56] and summarized in Table 4. The term “1st piece” refers to the portion of the specimen that meets the upper grip of the MTS during testing, primarily the section interacting with the nut. In contrast, the term “2nd piece” refers to the portion of the specimen that contacts the lower grip of the MTS during testing, the area in contact with the bolt.

Figure 8 shows the damaged sections of each specimen, as detailed in Table 4. The “1st Piece” is shown on the left and the “2nd Piece” on the right in the photographs. At w/D = 3.0, tension failure (L1I) was observed in each segment, yet the specimen maintained its overall form, as shown in Figure 8a. This failure pattern aligns with previous research, indicating unequal stress distribution due to inadequate internal contact between the bolt and hole sides during single-lap shear testing [24]. In Figure 8b, tension failure (L1I) and bearing failure (B1I) are observed at w/D = 4.0. As the load increases, damage initiates on one side of the composite laminate plate and propagates to the other. Figure 8c shows bearing failure (B1I) occurring in both sections at w/D = 6.0. When a composite laminate bolted joint fails, slight damage occurs on the opposite side of the laminate plates, leading to inadequate material performance. 

Thus, failure occurs before the load is transferred to the opposite piece, particularly noticeable at w/D = 3.0 when the specimen’s width is smaller than the hole size. While tension failure (L1I) occurs in the first segment, bearing failure (B1I) is evident in the second. This highlights the enhancement in stress-bearing capacity by widening the specimen w/D from 3.0 to 4.0 while maintaining the hole size at 0.250 inches. Bearing failure is detected in both specimen sections as the w/D ratio approaches 6.0, while tension failure (L1I) is only noticeable when the w/D ratio exceeds 6.0. Further testing with w/D ratios of 4.0 and 6.0 is necessary, as these results will significantly impact structural design decisions.

Bearing failure was consistently observed at a w/D ratio of 4.0, aligning with previous research findings [61,62,63,64,65,66,67,68]. Several studies have demonstrated variations in surface delamination depending on specific laminate configurations. Alaattin Aktas et al. provided insights into failure modes corresponding to the w/D ratio, with the e/D ratio fixed at 4.0: L1I failure occurred at w/D = 2.0, while bearing failure was evident at w/D = 4.0 [61,62,63]. Buket Okutan found that for e/D ratios greater than 3, bearing mode failure predominated, whereas at a value of 3, shear-out, a weaker failure mode, was observed [64]. Luigi Calabrese et al. indicated that bearing failure occurs when e/D is 1.5 and w/D is ≥4.0. However, this study was conducted on glass composite laminates and should be considered as reference material due to varied findings [65]. Luca Giorleo suggested a threshold of w/D = 4.5 for simultaneous bearing and tension failures [66]. Ramazan Karakuzu reported tension failure at w/D = 2 for an e/D ratio of 3.0 and bearing failure at w/D ≥ 3.0 [67]. Similarly, Sanjay Kumar reported tension failure at w/D = 2.0, mixed failure at w/D = 3.0, and bearing failure at w/D ≥ 4.0 [68].

Combining these varied findings, it is possible to infer that, while variances occur depending on material type, hole size, and reference e/D ratios, shear failure is often prominent for w/D ratios greater than 4.0. Consequently, more cautious criteria are re-quired due to potential faults in the manufacturing or drilling processes. Echoing similar trends to previous research, this comprehensive study offers significant insights into the quasi-isotropic ([+45/0/-45/90]_2S_) laminated specimens used. Specifically, for an e/D ratio of 3.0, a w/D ratio of 4.0 serves as a threshold, with tension failure occurring below and bearing failure occurring below.

Similarly, in Figure 8d, shear failure is observed in the first section of a specimen with an e/D ratio of 1.5. Figure 8e illustrates bearing failure at an e/D ratio of 2.0, while Figure 8f demonstrates an increase in length because of bearing failure at an e/D ratio of 3.0. These figures in Figure 8 clearly show various failure mechanisms, along with the corresponding length increase resulting from bearing failure after reaching the maximum load. Changes in the e/D ratio exhibit a similar pattern to changes in the w/D ratio and the resulting differences in specimen failure mechanisms. At an e/D ratio of 1.5, specimens experience failure like that observed at a w/D ratio of 3.0, indicating failure before load transmission to the second piece. As the e/D ratio increases to 2.0, specimens experience failure in the first section and bearing failure in the second.

As the e/D ratio approaches 3.0, both parts of the specimen experience bearing failure, indicating the absence of tension failure (L1I). This result aligns with findings from prior investigations [69,70,71,72,73,74]. Alaattin Aktas reported that, while adjusting the e/D ratio from 1.0 to 5.0 with a fixed w/D ratio of 4.0 for CFRP plates, shear and bearing failures occurred together when the e/D ratio exceeded 5.0, suggesting a significantly conservative value compared to the e/D ratio of 2.0 presented in this study. However, since this study fixed w/D at 6.0, differences in these results were observed [69,70]. Buket Okutan proposed an e/D ratio of 2.0, which aligns with the threshold value of 2.0 found in this study. However, since this is based on experimental data for glass laminates, it can be utilized as a reference material [71]. B. Okutan Baba conducted experiments considering various fiber orientations and laminate geometries, reporting that bearing failure occurred when the e/D ratio was 5 for w/D = 5 in approximately [90/±45]s orientations and when the e/D ratio was above 4.0 for [90/0/90]s orientations [72].

In this study, it was found that shear failure occurs for e/D ratios below 2.0 and bearing failure occurs for ratios above 2.0. However, the variance in these threshold values, like the discussion on w/D threshold, can be influenced by several factors. Additionally, the reason why this value was relatively low at 2.0 in this study is because the w/D was fixed at 6.0. Therefore, the main insight proposed in this study is as follows: while echoing similar trends to prior research, in specimens laminated quasi-isotropic ([+45/0/-45/90]_2S_) with w/D at 6.0, an e/D ratio of 2.0 serves as a threshold. Below this level, shear failure occurs, while above it, bearing failure occurs.

## 5. Conclusions

The study investigated the mechanical characteristics of mechanically coupled composite joints. The differences in width-to-diameter (w/D) and edge-to-diameter (e/D) ratios had a substantial influence on the specimens’ final bearing strengths. This comparative study demonstrated that changing the w/D and e/D ratios resulted in significant decreases in ultimate bearing strengths, which is consistent with previous research. Furthermore, the study carefully examined the bearing strength and failure mechanisms of specimens with different w/D and e/D ratios. Notably, increasing the w/D ratio resulted in an increase in bearing strength, moving the failure mechanism from tension and shear to bearing failure. Furthermore, shear failure of the fastener’s thread was regularly seen throughout all samples.

The findings of this study provide crucial basic data for reducing structural damage in the design of mechanically connected composite CFRP structures. Specifically, it was determined that maintaining a w/D ratio of at least 6.0 and an e/D ratio of at least 3.0 is critical for maximum performance and stability. A thorough grasp of how CFRP structures behave under various loading circumstances and failure modes is critical. Such knowledge enables educated decision-making in material selection, component design, and overall structural integrity, improving the dependability and efficacy of designed systems.

## Figures and Tables

**Table 1:** Dimensions of the Test Specimens (Dimension: mm).

Ratio	Width	Edge Distance	Thickness	Diameter
w/D = 3.0	19.07 ± 0.03	19.05 ± 0.01	2.19 ± 0.02	6.36 ± 0.01
w/D = 4.0	25.38 ± 0.02	19.05 ± 0.01	2.20 ± 0.03	6.36 ± 0.02
w/D = 6.0	38.11 ± 0.01	19.05 ± 0.02	2.19 ± 0.02	6.36 ± 0.01
e/D = 1.5	38.04 ± 0.01	9.51 ± 0.03	2.21 ± 0.01	6.36 ± 0.01
e/D = 2.0	38.02 ± 0.02	12.72 ± 0.01	2.19 ± 0.02	6.36 ± 0.01
e/D = 3.0	38.07 ± 0.01	19.05 ± 0.02	2.19 ± 0.02	6.36 ± 0.01

**Table 2:** Comparison of bearing strength for each w/D ratio (w/D = 3.0, 4.0, and 6.0).

Ratio	2% Offset Bearing Strength (MPa)	Ultimate Bearing Strength (MPa)
w/D = 3.0	548 ± 45	664 ± 7
w/D = 4.0	730 ± 29	859 ± 31
w/D = 6.0	789 ± 46	949 ± 54

**Table 3:** Comparison of bearing strength for each e/D ratio (e/D = 1.5, 2.0, and 3.0).

Ratio	2% Offset Bearing Stress (MPa)	Ultimate Bearing Stress (MPa)
e/D = 1.5	668 ± 25	721 ± 27
e/D = 2.0	774 ± 13	901 ± 47
e/D = 3.0	789 ± 46	949 ± 54

**Table 4:** Failure modes of specimens according to w/D and e/D ratios.

Ratio	1st Piece	2nd Piece	Fastener
w/D = 3.0	L1I	-	S3T
w/D = 4.0	L1I + B1I	B1I	S3T
w/D = 6.0	B1I	B1I	S3T
e/D = 1.5	S1I	-	S3T
e/D = 2.0	S1I + B1I	B1I	S3T
e/D = 3.0	B1I	B1I	S3T

## Data Availability

Data are contained within the article.
